# Resilience of cassava (*Manihot esculenta* Crantz) to salinity: implications for food security in low-lying regions

**DOI:** 10.1093/jxb/erw302

**Published:** 2016-08-09

**Authors:** Ros Gleadow, Amelia Pegg, Cecilia K. Blomstedt

**Affiliations:** School of Biological Sciences, Monash University, Clayton, Melbourne, Victoria 3800, Australia

**Keywords:** Cassava, cyanogenic glucosides, food security, Konzo, linamarin, salinity, sea level.

## Abstract

Rising sea levels are threatening agricultural production in coastal regions due to inundation and contamination of groundwater. The development of more salt-tolerant crops is essential. Cassava is an important staple, particularly among poor subsistence farmers. Its tolerance to drought and elevated temperatures make it highly suitable for meeting global food demands in the face of climate change, but its ability to tolerate salt is unknown. Cassava stores nitrogen in the form of cyanogenic glucosides and can cause cyanide poisoning unless correctly processed. Previous research demonstrated that cyanide levels are higher in droughted plants, possibly as a mechanism for increasing resilience to oxidative stress. We determined the tolerance of cassava to salt at two different stages of development, and tested the hypothesis that cyanide toxicity would be higher in salt-stressed plants. Cassava was grown at a range of concentrations of sodium chloride (NaCl) at two growth stages: tuber initiation and tuber expansion. Established plants were able to tolerate 100mM NaCl but in younger plants 40mM was sufficient to retard plant growth severely. Nutrient analysis showed that plants were only able to exclude sodium at low concentrations. The foliar cyanogenic glucoside concentration in young plants increased under moderate salinity stress but was lower in plants grown at high salt. Importantly, there was no significant change in the cyanogenic glucoside concentration in the tubers. We propose that the mechanisms for salinity tolerance are age dependent, and that this can be traced to the relative cost of leaves in young and old plants.

## Highlights

Cassava tolerates low but not high levels of salinity via ion exclusion and higher foliar cyanide. Mechanisms for resistance are related to the decreasing value of leaves as plants mature.

## Introduction

Rising sea levels and salinization of groundwater is becoming an increasing problem in many parts of the world, reducing the area of land available for agricultural crops (Church *et al.*, 2013; Nunn, 2013). Globally, one-fifth of irrigated land is salt affected (450 000 km^2^), resulting in huge losses in productivity (Munns and Gillman, 2015). The ablity to tolerate salt varies within and between species. Barley (*Hordeum vulgare*), for example, is one of the more salt-tolerant crops, while lupins (*Lupinus albus*) is one of the most sensitive (Munns, 2002). Little is known about the impact of salinity on some of the world’s major staples.

Cassava (*Manihot esculenta* Crantz) is the most widely grown root crop in the world and the tuberous roots are the principle source of calories for many of the world’s poorest people (Nweke *et al.*, 2002; FAO, 2014). Cassava has the ability to cope with a wide range of environmental stresses and continues to produce tubers under poor growing conditions, such as low nutrients and drought (El-Sharkawy, 1993; Jørgensen *et al.*, 2005; Burns *et al.*, 2010), yet little is known about how it responds to salt stress. Its classification as ‘moderately sensitive’ by the FAO is based on three early studies (Anon, 1976; Hawker and Smith, 1982; Indira, 1978). More recently, Carretero *et al.* (2008), in a study of pre-tuberous plants, found large effects on growth at 68mM sodium chloride (NaCl) and only 30% survival at the highest concentration (136.8mM NaCl).

Even less is known about the impact of salinity on the nutritional value of cassava. Unlike all other staple foods, cassava can be lethal if not correctly processed due to the high levels of the cyanogenic glucosides, linamarin and lotaustralin, that break down to release cyanide when mixed with specific β-glucosidases (McMahon *et al.*, 1995; McKey *et al.*, 2010). Consumption of cassava that is high in cyanide may cause diseases in humans, such as Konzo (an irreversible paralysis of the lower limbs), tropical ataxia, and even death (Cliff *et al.*, 1997; Burns *et al.*, 2010; Bradbury *et al.*, 2011). There is a strong link between Konzo epidemics and the increase in cyanide concentrations during droughts (Ernesto *et al.*, 2002), but there are no published studies on whether salinity-induced physiological droughts also result in higher levels of cyanide, and possible threats to health and food security. Ballhorn and Elias (2014), in the only published study on the effect of salinity on the production of cyanogenic glucosides, found for *Trifolium repens* that the cyanogenic glucoside concentration did increase in proportion to increasing salinity. Salinity is also known to affect the micronutrient concentration in plants. Given that children consuming cassava as a staple food are at risk for micronutrient deficiencies, particularly zinc, iron, and vitamin A (Gegios *et al.*, 2010), it is important to assess the possible impact of salinity on their availability in cassava food products.

Cyanogenic glucosides are an effective defence again herbivores (Gleadow and Woodrow, 2002), but they represent a significant drain on resources that might otherwise be used for growth (e.g. Simon *et al.*, 2010). This is balanced by the alternative roles they may play in primary metabolism, such as the transport and storage of nitrogen and mitigation of oxidative stress (Gleadow and Woodrow, 2002; Kongsawadworakul *et al.*, 2009; Selmar and Kleinwächeter, 2013; Gleadow and Møller, 2014). Thus the partitioning of resources is a balance between defence, particuarly when growth is limited, and, for example, stress tolerance and nitrogen turnover.

The aim of this study was, therefore, to determine the effect that salinity had on biomass and nutritional composition at two different life stages of cassava. We tested the tolerance of established plants, with well-developed tubers, to a wide range of NaCl solutions. The second experiment involved a detailed study on young clonally propagated plantlets through to tuber initiation. Photosynthetic parameters, growth indices, mineral nutrient composition, and cyanogenic glucoside concentration were determined and used to estimate the impact of salinity on plant production and nutritional value.

## Materials and methods

### Plant material and growing conditions

Cassava (*Manihot esculenta* Cranz) cv. MAus7 was grown in a greenhouse at the School of Biological Sciences complex Monash University, Clayton. Average temperature was 25 °C/23.5 °C day/night with a relative humidity of 75%. Glasshouses received natural light. The photoperiod was extended to 14h from May to September 2014 using sodium lamps (MK-1 Just-a-shade, Ablite Australia, Sunnfield Enterprises, Allambie Heights, NSW, Australia). Plants were rotated weekly to reduce microenvironment effects.

#### Experiment 1: effect of a range of salt concentrations on established cassava plants

The effect of salt on cassava plants with established tuberous roots (‘tubers’, hereafter) was tested. Cassava (one plant per pot) was grown in 8 litre pots containing commercial potting mix with slow-release fertilizer (Osmocote^®^) for 8 months (June 2013–January 2014) and then treated with four different concentrations of NaCl for 4 weeks. Eight-month-old cassava plants were matched according to height and leaf number to form five sets with seven plants in individual pots in each group. One group was harvested for initial biomass determination (*n*=7 plants). The other groups were assigned to one of four different salt treatments (0, 50, 100, or 150mM NaCl) (Hawker and Smith, 1982; Carretero *et al.*, 2008) in a randomized matched-pair design. Plants were watered with 1.4 litres of solution two times per week, such that the solution ran through the pots. Pots were flushed weekly with 2 litres of water to prevent the build-up of salt in the soil. All plants were destructively harvested after 28 d for biomass determination and chemical analysis.

#### Experiment 2: long-term effects of salinity on growth and tuber initiation

In the second experiment, longer term effects of salt on plants prior to tuber initiation were tested. In this experiment, young plants were established from cuttings (January 2014) and transplanted after 2 months (~15cm tall, three leaves) into 2 litre pots containing potting mix (as above) and mixed 5:1 (v/v) with soil containing mycorrhizal colonies, from the Jock Marshall Reserve, Monash University following Vandegeer *et al.* (2013). During this time, they were watered twice a week, once with a commercial full nutrient solution (Aquasol^®^, 10ml l^−1^ H_2_O), and once with plain water. Before treatments were imposed, a set of 10 plants were harvested for dry mass determination. Plants were then watered with three different concentrations of salt [0, 40, and 80mM NaCl (*n*=10) based on the results of Experiment 1]. In order to avoid a shock response, the salt concentration was increased gradually, starting at 20mM and increasing by 20mM every 3 d until the levels of 40mM and 80mM were reached. These levels were then maintained for the remainder of the experiment, a total of 70 d. The plants were watered with 300ml of their appropriate solution twice a week. This volume was enough to saturate the soil. Pots were flushed weekly with 500ml of water to prevent accumulation of salt in the soil. To ensure plants did not become nutrient limited, they were watered with Aquasol^®^ on days 49, 52, and 56 of the study at a rate of 10ml l^−1^.

The stem height and leaf number of each plant were recorded each week. Stem height was measured from the soil surface to the point where the newest leaf was expanding. The number of lobes of the third fully expanded leaf and the length of the middle lobe of that leaf was also recorded weekly. The lobe length was the distance from the point where the leaf blade was attached to the petiole to the tip of the lobe. All plants (*n*=30) were destructively harvested after 70 d (see below).

### Harvesting protocol

The biomass of plants was separated into above-ground (stem, petioles, leaves) and below-ground (cutting, roots, tubers). The above-ground biomass of plants was removed by cutting the stem at the soil surface. Leaves were removed from the plant and separated into three classes: fully expanded, unexpanded, or senescent. Expanded leaves were defined as fully developed leaves, unexpanded leaves were young, soft leaves, and senescent leaves were defined as leaves that were yellow or brown in colour on >50% of the leaf surface. Leaf fresh weight was recorded for all classes and leaf area was taken for expanded and unexpanded leaves using a leaf area meter (LI-3000 Portable Area Meter, Li-Cor, Lincoln, NE, USA). Three leaf discs (diameter=5mm) were taken from the third fully expanded leaf and one disc from tubers and frozen at –20 °C for later analysis for cyanogenic glucosides. Height, and stem and petiole weight were recorded. Roots and tubers were washed and all soil removed where possible. For Experiment 1, tubers and roots were weighed separately, oven-dried at 60 °C for 14 d, and dry weights determined. For Experiment 2, roots were separated into fine roots and thick roots (tuberous roots). Thick roots were defined as roots that were white in appearance and >2mm in diameter. All leaf and root tissue was frozen in liquid nitrogen and freeze-dried for 5 d, and weighed. The remaining biomass (stems and petioles) was oven-dried at 60 °C for 14 d and dry weights determined.

### Growth indices

The root:shoot ratio was calculated by dividing the total below-ground biomass (DW) by the total above-ground biomass (DW). The relative growth rate (RGR) was calculated as follows: RGR=log(W_2_)–log(W_1_)/(*t*_2_–*t*_1_), where W_1_ and W_2_ are the initial and final dry mass at time 1 (*t*_1_) and time 2 (*t*_2_). The specific leaf area (SLA) of fully expanded leaves was calculated as leaf area per g dry leaf mass.

### Photosynthetic parameters: assimilation rates, *F*_v_'/*F*_m_', greenness, and chlorophyll

Light-saturated measurements of the photosynthetic rate and light-adapted chlorophyll fluorescence, *F*_v_'/*F*_m_', were made using a Li-Cor 6400 gas exchange system on all living plants in Experiment 2 in the week before harvesting (20 August 2014). Three concordant measurements were made on the third fully expanded leaf from each living plant at 700 µmol m^−2^ s^−2^ PAR; 400ppm CO_2_, 25 °C (growth temperature), and a relative humidity of 50%, following Gleadow *et al.* (2009).

Total chlorophyll concentration was measured following Gleadow *et al.* (1983) as modified by Burns *et al.* (2002). Freeze-dried leaf tissue (100mg) was extracted twice in 2ml of 80% acetone and the absorbance measured at 647, 665, and 750nm using a FLUOstar Galaxy plate reader (BMG, Australia). Greenness measurement readings were made of the third fully expanded leaf using the GreenIndex+ Ap (Spectrum Technologies Inc.^®^) by taking a photograph of the middle lobe against a background of known colour and using in-built algorithms. To our knowledge, this is the first time GreenIndex+ measurements have been made on cassava.

### Plant composition: cyanogenic glucosides, total nitrogen, and micro- and macronutrients

The cyanogenic glucoside concentration was measured by the evolved cyanide method. Freeze-dried tissue (three leaf discs diameter=1cm^2;^ or one tuber disc, 2mm thick) was placed into sealed vials with 270 µl of 0.1M pH 6.4 phosphate buffer, and a separate 0.2ml inner tube containing 200 µl of 1M NaOH. Vials were then frozen and thawed at room temperature twice to disrupt the cells, and incubated for ~19h at 37 °C. The cyanide evolved from the cyanogenic glucosides trapped in the NaOH was assayed colorimetrically following Blomstedt *et al.* (2012). To ensure complete conversion of the cyanogenic glucosides to cyanide, latex [30 µl, in 0.1M phosphate buffer pH 6.4, 1:100 (v/v)] was added to the reaction vial, as latex contains the degradative β-glucosidases and α-hydoxynitrile lyases necessary to degrade both linamarin and lotaustralin (Jørgensen *et al.*, 2005). Tissue in vials was rinsed, dried, and weighed for biomass determination to allow the calculation of the hydrogen cyanide potential (HCNp) of the tissue analysed.

Total elemental nitrogen and carbon were measured on finely ground, freeze-dried leaf and tuber samples using a LECO CNS2000 analyser. The remaining nutrient analyses were performed using inductively coupled plasma (ICP)-MS on microwave-digested samples (Environmental Analysis Laboratory, Southern Cross University, NSW, Australia).

### Statistical analysis

Data were analysed with Prism Graphpad 6^®^ using ANOVA and *t*-tests. Matched-pair ANOVAs were used for analysis of Experiment 1 as plants were grouped according to size. Means were compared using post-hoc Tukey’s tests (*P*<0.05) where a statistical significance was detected between groups. Data were log transformed if required in order to satisfy the assumptions of normality.

## Results

### Experiment 1: effect of a range of salt concentrations on established cassava plants

**Fig. 1.**
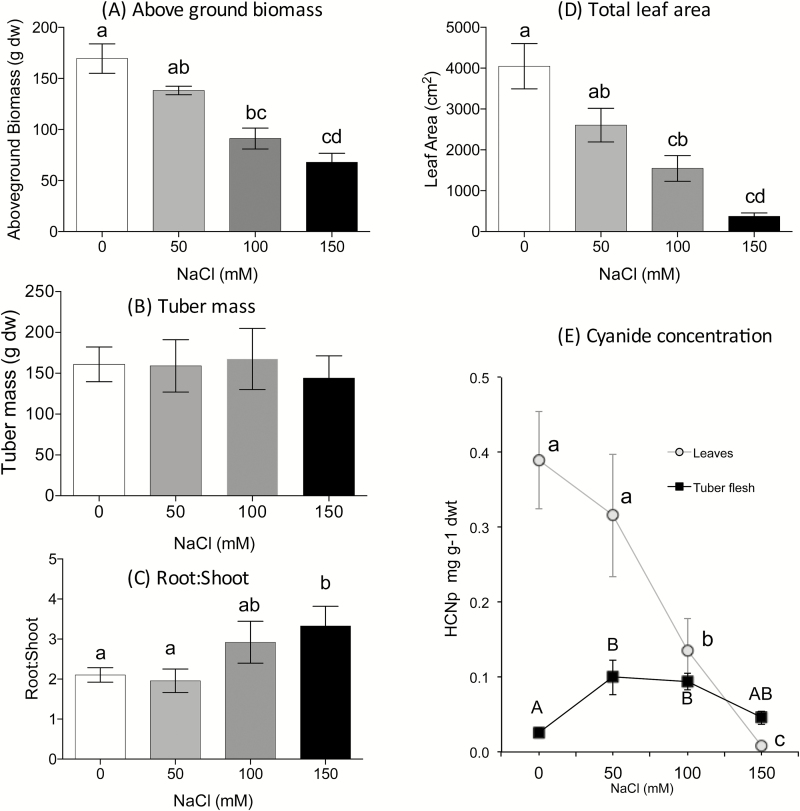
Biomass, growth measurements, and tissue hydrogen cyanide potential (HCNp) of 6-month-old cassava plants grown at 0, 50, 100, and 150mM NaCl for 28 d (Experiment 1; *n*=7 for each treatment). (A) Above-ground biomass; (B) tuber biomass; (C) root:shoot ratio; (D) total leaf area; (E) foliar and tuber HCNp. Means (±SE) with different letters are significantly different at *P*<0.05.

Plants were grown at four concentrations of salt for 28 d. Above-ground biomass decreased with increasing salinity and was significantly lower in plants grown at 100mM and 150mM NaCl (Fig. 1A) compared with controls. Mean above-ground dry weight in control plants was 169±14.5g, but was only 67.9±8.9g in plants grown at 150mM NaCl (*F*_3,24_=20.35, *P*<0.0001; Fig. 1A). The root:shoot ratio was higher as a consequence of increased salinity, driven by the difference in above-ground biomass, as there was no significant difference in tuber biomass between treatments (*F*_6,18_=3.7, *P*=0.85; Fig. 1B, C). Leaf area of plants decreased as the salinity level increased, primarily through plants shedding leaves, with significant differences detected between control and 100mM, control and 150mM, and 50mM and 150mM NaCl treatment groups (*F*_3,24_=16.64, *P*=0.021, 0.003, and 0.005, respectively; Fig. 1D).

The HCNp in the leaves decreased with increasing salt concentration (Fig. 1E). In the tubers, HCNp initially increased and was significantly higher at 50mM and 100mM NaCl (*F*_3,22_=6.46, *P*=0.006 and 0.015, respectively). At the highest salt treatment, the HCNp decreased in the tubers, but there was no significant difference compared with any other treatment.

### Experiment 2: long-term effects of salinity on young cassava plants and tuber initiation

#### Plant growth, biomass, and phenology

**Fig. 2.**
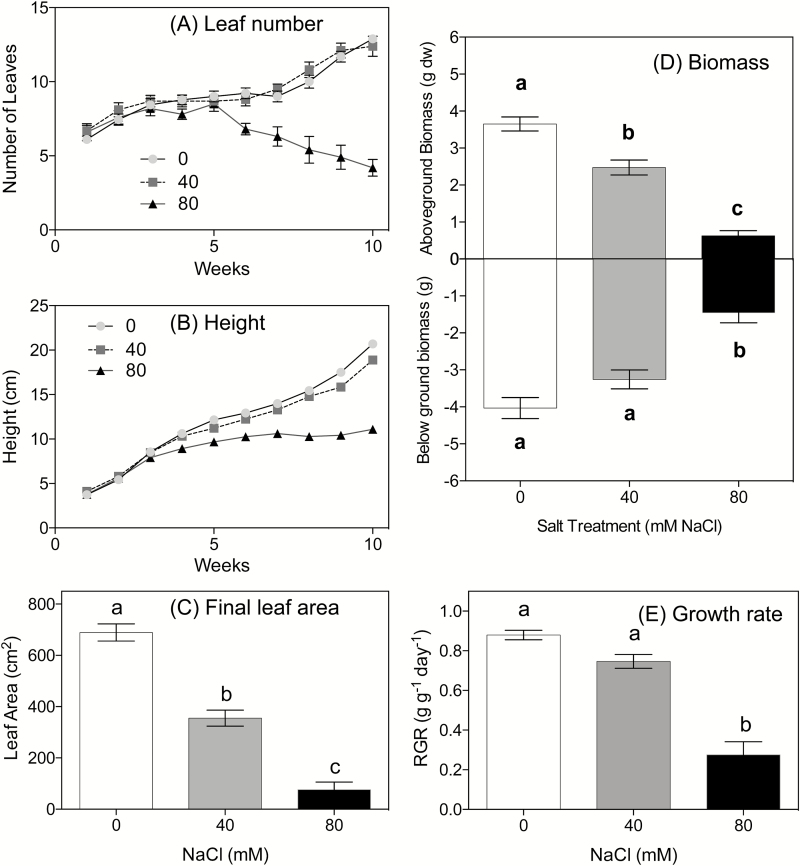
Phenology and final size of cassava plants grown for 9 weeks with nutrient solutions containing different concentrations of salt (0, 40, and 80mM NaCl) (Experiment 2; *n*=10 for each treatment). (A) Leaf number and (B) height of plants measured weekly; (C) final total leaf area (all leaf classes); (D) total above- and below-ground biomass; (E) relative growth rate. Values are means (±SE) of 10 replicates. Columns with different letters represent significant differences at *P*<0.05 (Tukey’s test).

The number of leaves present on each plant was recorded once a week for the duration of the study. After 5 weeks, plants in the 80mM NaCl group began to lose leaves at a steady rate, whereas plants in the control and 40mM NaCl groups had steady increases in their number of leaves over the course of the experiment (Fig. 2A). Six of the 10 plants grown at 80mM NaCl died (i.e. all leaves abscised and there was no subsequent sign of recovery), four at day 56 and the other two at day 60 of the study. Surviving plants were shorter (Fig. 2B), had fewer fully expanded leaves, and had more senescent leaves than plants grown at 40mM NaCl (Fig. 2; Supplementary Table S1 at *JXB* online). There was a significant decrease in leaf area of plants grown at both 40 mM and 80mM NaCl (Fig. 2C). Total biomass was highest in plants grown without salt, with an average mass of 7.68±0.42g compared with the plants grown at 40mM (5.73±0.32g) and 80mM NaCl 2.09±0.43g, respectively (*F*_2.27_=52.24, *P*<0.0001; Fig. 2D). Control plants had greater above-ground biomass than plants from either of the salt treatments (*F*_2.27_=72.40, *P*<0.0001, Fig. 2D), but the below-ground biomass was not significantly different between the control and 40mM NaCl groups. Thus, the higher root:shoot ratio in the 80mM plants of 2.98±0.64 compared with 1.33±0.08 in the 40 mM-grown plants (Supplementary Table S1; *F*_2,27_=7.58, *P*=0.0038) was driven primarily by the lower above-ground biomass, as the fine root mass was small and only one plant produced a tuber (Fig. 2D; Supplementary Table S1). There was no significant difference in the RGR between plants grown at 0mM and 40mM NaCl, but there was a significant decrease in plants at 80mM salt (Fig. 2E).

#### Photosynthetic parameters: assimilation rate, stomatal conductance, *F*_v_'/*F*_m_', and chlorophyll

**Fig. 3.**
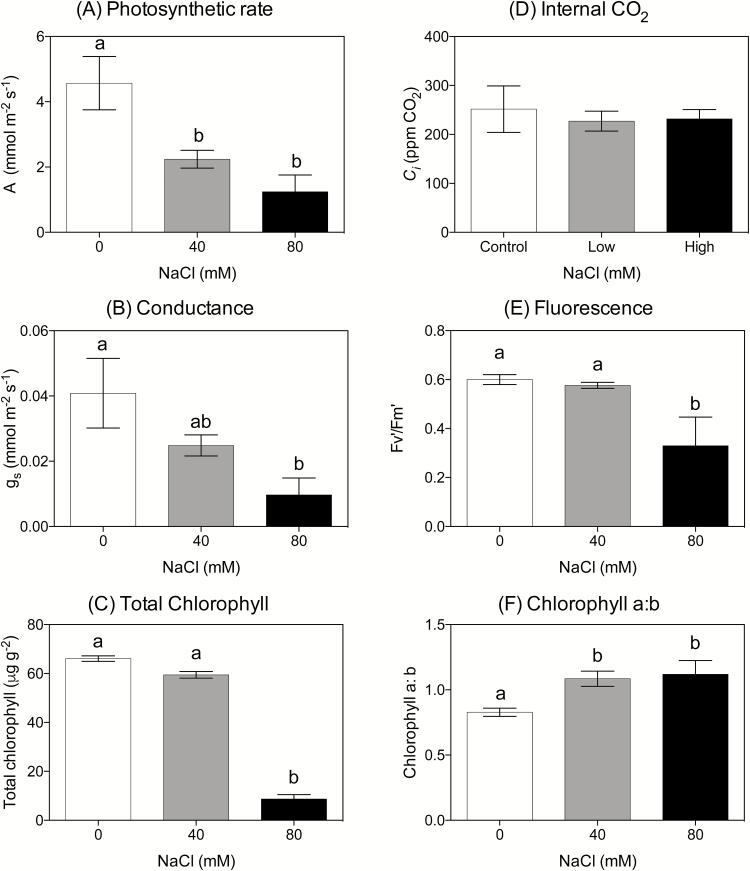
Photosynthetic parameters for young cassava plants grown at 0, 40, and 80mM NaCl treatments for 9 weeks (*n*=10 except at 80 mM where *n*=4.) (A) Carbon assimilation (*A*); (B) leaf conductance (*g*_s_); (C) total chlorophyll; (D) internal CO_2_ concentration (*C*_i_); (E) light-adapted fluorescence (*F*_v_'/*F*_m_'); (F) Chl *a*/Chl *b* ratio. Means (±SE) with different letters represent significant differences at *P*<0.05 (Tukey’s test).

Assimilation, stomatal conductance, and *F*_v_'/*F*_m_' were measured on the third fully expanded leaf. The assimilation rate of control plants was 4.57±0.82 mmol m^−2^ s^−1^, 2-fold greater than that of plants grown at 40mM (2.24±0.27 mmol m^−2^ s^−1^) and nearly 5-fold higher than plants from the highest 80mM (1.24±0.51 mmol m^−2^ s^−1^) salt treatment (*F*_2,21_=7.53, *P*=0.0182 and 0.0055, respectively; Fig. 3A). Stomatal conductance followed a similar pattern to that of the photosynthetic rate; however, there was only a significant difference between control (0.04±0.01 mmol m^−2^ s^−1^) and 80 mM- (0.01±0.005 mmol m^−2^ s^−1^) treated plants (*F*_2,21_=3.54, *P*=0.0420; Fig. 3B). Internal CO_2_ concentrations were similar in all treatments (Fig. 3D). There was no significant difference in *F*_v_'/*F*_m_' between control and 40mM plants, with an overall mean of 0.59±0.01. *F*_v_'/*F*_m_' was much lower in plants grown under 80mM NaCl, with a mean of 0.33±12 (*F*_2,21_=8.77, *P*=0.0017), indicating severe stress (Fig. 3E).

Total chlorophyll concentration also decreased with increasing salt concentration. Plants grown under 40mM NaCl had a lower chlorophyll concentration than control plants but the difference was not significant (F_2,23_=411.10, *P*=0.0036; Fig. 3C) and the concentration of chlorophyll in leaves from 80 mM-grown plants was significantly lower than that of both the control plants and plants at 40mM NaCl (*F*_2,23_=411.10, *P*<0.0001). The Chl *a*/*b* ratio was significantly higher for 40mM and 80mM plants compared with control plants (*F*_2,23_=7.23, *P*=0.0095 and 0.0110, respectively; Fig. 3F). Measurement of leaf greenness was to approximate total chlorophyll concentration, with a highly significant Pearson’s correlation coefficient of 0.67 (*P*<0.001) (Supplementary Table S1).

#### Cyanogenic glucoside and nutrient analysis

**Fig. 4.**
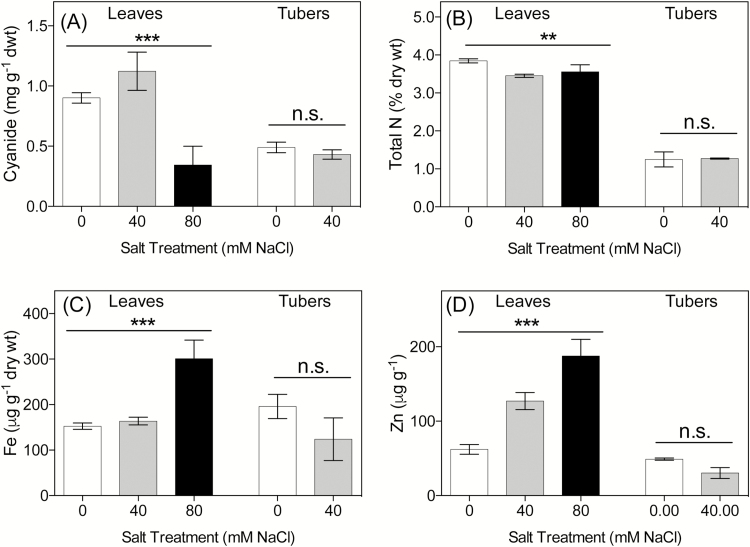
Concentration of (A) cyanogenic glucosides (measured as evolved cyanide, HCNp), (B) total elementary nitrogen, (C) iron (Fe), and (D) zinc (Zn) of leaves and tubers of young cassava plants after 9 weeks treatment with nutrient solution containing three different concentrations of salt. No tubers were present in the 80mM NaCl treatment. Values are means ±SE. *n*=10, except at 80mM NaCl where final *n*=4 for analysis. Significant differences are indicated on the line over each group of means. ***P<0.001; **P<0.01; n.s.=not significant. A full list of macro- and micronutrients is given in Table 1.

Cyanide assays were performed on freeze-dried leaf and tuber tissue. HCNp was highest in the leaves of plants grown at 40mM, with lower levels in control plants and those grown at 80mM NaCl (*F*_2,26_=9.32, *P*=0.0149 and 0.0008, respectively, Fig. 4A). There was no difference between control and 40mM groups in the amount of HCNp found in tubers (Fig. 4A). Statistical tests on HCNp were unable to be performed for tubers grown at 80mM NaCl as only one plant grown at this salt level contained tuberous roots. Overall, there was no significant difference in the HCNp:total biomass ratio between treatments (*F*_2, 27_=0.43, *P*=0.6536), indicating that observed changes in HCNp were not driven by changes in biomass.

Total nitrogen concentration was higher in the leaves of control plants compared with 40mM plants (*F*_2,22_=8.14, *P*=0.0019; Table 1; Fig. 4B), although no difference between control and 80mM plants or 40mM and 80mM plants was found. Carbon was lower in leaves of 80 mM-grown plants so there was no overall difference in the C:N ratio between control and 80 mM-grown plants. However, the leaves of plants grown at 40mM NaCl had a higher C:N ratio compared with control and 80mM plants (*F*_2,22_=14.86, *P*=0.0014 and 0.0002, respectively; Table 1).

**Table 1.** Nutrient analysis of leaves and tubers of cassava plants (*n*=4) grown at three concentrations of salt (0, 40, and 80mM NaCl)

	Leaves			Tubers	
Nutrient\NaCl	0 mM	40 mM	80 mM	0 mM	40 mM
N (% DW)	3.85±0.06 a	3.45±0.04 b	3.56±0.19 ab	1.250±0.198	1.273±0.013
C (% DW)	44.64±0.09 a	43.85±0.20 a	39.32±1.83 b	41.13±0.20	40.63±0.15
C:N	11.63±0.17 a	12.74±0.19 b	11.08±0.34 a	34.80±6.17	31.90±0.36
Na (% DW)	0.01±0.00 a	0.02±0.01 a	1.78±0.72 b	0.04±0.02 a	0.80±0.05 b
K (% DW)	2.96±0.07	2.82±0.07	2.88±0.11	1.14±0.07 a	1.60±0.08 b
P (% DW)	0.30±0.01 a	0.31±0.01 a	0.39±0.02 b	0.02±0.00	0.22±0.03
S (% DW)	0.32±0.01 a	0.30±0.01 b	0.44±0.09 a	0.51±0.33	0.15±0.03
Mg (% DW)	0.24±0.01 a	0.31±0.01 b	0.38±0.03 c	0.18±0.01	0.15±0.05
Mn (mg kg^–1^)	42.70±1.79 a	84.80±5.56 b	107.00±6.09 c	23.33±1.76	14.00±6.43
Cu (mg kg^–1^)	3.30±0.21 a	4.20±0.20 a	7.00±1.00 b	6.67±0.33	6.33±3.93
Zn (mg kg^–1^)	68.40±2.20 a	135.70±8.31 b	209.20 c	49.00±1.53	30.33±7.36
Fe (mg kg^–1^)	152.60±6.88 a	163.80±8.49 a	300.80±41.13 b	196.00±26.54	124.00±46.97
B (mg kg^–1^)	51.60±1.54	59.50±2.54	53.60±4.41	9.00±0.58	6.33±3.53
Mo (mg kg^–1^)	0.29±0.02	0.57±0.26	1.96±1.39	0.57±0.12	0.37±0.17
Co (mg kg^–1^)	0.10±0.00 a	0.11±0.01 a	0.24±0.04 b	0.23±0.03	0.13±0.03
Si (mg kg^–1^)	528.90±31.11	451.40±21.24	562.00±31.16	310.00±21.70	239.70±40.27

Means (±SE) followed by different letters within tissue types are significantly different at *P*<0.05 (Tukey’s test).

Analysis of leaves (all treatments) and tubers (0 and 40mM NaCl treatment only due to lack of tuber initiation at 80mM NaCl) showed that there were significant differences in concentrations of key nutrients and trace elements between treatments (Table 1; Fig. 4C, D). The most striking difference is seen in the concentration of sodium. Plants at 80mM NaCl had higher amounts of sodium in their leaves (1.80%) compared with control (0.01%) and 40mM (0.02%) plants (*F*_2,22_=50.58, *P*<0.0001 and 0.0073, respectively; Table 1). The sodium concentration in the tubers in plants grown at 40mM NaCl was nearly 20 times higher than in control plants (*P*=0.0022). The amount of potassium was also significantly higher in the tubers of plants grown at 40mM salt, but the magnitude of the increase was much less, with a 1.4-fold increase (Table 1).

Iron and zinc concentrations were significantly higher in leaves of plants grown at 80mM NaCl (Fig. 4C, D). Concentrations of these two nutrients were somewhat lower in the tubers of salt-stressed plants, but the differences were not significant. Of the remaining macronutrients, the concentration of manganese in leaves also increased with rises in salinity levels and ranged from 43mg kg^−1^ in control plants to 107mg kg^−1^ in 80mM plants (*F*_2,22_=47.13, *P*<0.0001, <0.0001, and 0.0146, respectively; Table 1). The amount of magnesium increased with increasing NaCl concentration in leaf tissue, and was significantly different between all treatments. Leaf phosphorus was marginally higher in the leaf tissue of plants grown at 80mM NaCl and also 10-fold higher in the tubers of 40mM plants (Table 1). Leaf sulphur levels in 40mM plants were lower than in control and 80 mM-grown plants. The levels of trace elements in addition to iron and zinc, such as copper and cobalt, increased in leaf tissue with increasing salt (Table 1). In general, salt stress results in an increase in the nutrient levels in leaves and a decrease in the tubers (Table 1).

## Discussion

The impact of salinity on growth and nutritional value (i.e. the cyanogenic glucoside and micronutrient concentrations) depended on the age of the plant. Older plants that had already developed tubers were more salt tolerant than younger, pre-tuberous plants in terms of survival and growth. The key effect of salinity on cassava was a reduction in biomass, leaf area, and photosynthetic rate. There was an increase in HCNp in the leaves of young cassava plants under moderate stress (Fig. 4), but in the leaves of mature cassava plants, HCNp decreased step-wise with increases in salinity (Fig. 1). The age-affected differences may be related to: (i) the propensity for cassava to shed leaves in response to abiotic stress; (ii) the relatively high costs involved in excluding sodium; and (iii) relatively higher investment by younger plants in leaves compared with older plants.

### Growth and photosynthesis

We found that tuberous plants were able to tolerate fairly high concentrations of salt, up to 150mM NaCl (Fig. 1). In contrast, growth and survival of pre-tuberous plants was severely retarded at 80mM NaCl, with only one plant developing a tuber (Fig. 2). In both experiments, there was a decrease in mass of ~1% for every 1mM NaCl increase, relative to the initial biomass (*y*=–1.0197*x*+456.6; *R* ^2^=0.20; Experiment 1; Supplementary Fig. S1) and (*y*=–0.0699*x*+7.966; *R* ^2^=0.77; Experiment 2; Supplementary Fig. S2). These results are broadly consistent with earlier studies that report severe stunting and death of cassava plants between 50mM and 135mM, depending on variety, length of treatment, and soil environment, with older plants and those with mycorrhizae generally being more tolerant (Indira, 1978; Hawker and Smith, 1982; Carretero *et al.*, 2008).

The large reduction in biomass in both pre- and post-tuberous plants can be attributed to the loss of photosynthetic area and reduced rates of carbon assimilation. Weekly phenological measurements allowed us to track individual plants over the entire treatment period. In young plants, there was no significant reduction in leaf number until nearly 8 weeks in plants grown at 40mM NaCl and even in the 80mM treatment leaves were retained until 4 weeks after treatments commenced. This is consistent with the characteristic shedding of older leaves when plants are under stress, for example when droughted (Setter and Fregene, 2007; Vandegeer *et al.*, 2013). Final harvest data showed that leaf area was even more sensitive to salt than leaf number, with a significant reduction in pre- and post-tuberous plants at 40mM and 100mM NaCl, respectively, (Figs 2 and 1, respectively).

The concentration of chlorophyll, greenness, and *F*_v_'/*F*_m_' (measured on the third fully expanded leaf) were not significantly lower in plants grown at 40mM, indicating that these leaves were not under severe stress. Plants from the 80mM treatment, on the other hand, did show signs of stress with a lower *F*_v_'/*F*_m_', and decreased chlorophyll concentration. Vandegeer *et al.* (2013) showed that the health of these highly productive, expanded leaves is maintained well after drought has affected other parts of the plant metabolism, and leaves are only discarded after prolonged drought. The significant leaf loss after 4 weeks growth and the reduction in photosynthetic capacity suggest that at 80mM salt cassava is severely stressed.

### Nutrient analysis and evidence for ionic exclusion at low salinity

Evidence that cassava is able to tolerate low to moderate concentrations of salt comes from the ionic composition of the tissues. Foliar sodium concentration was the same at 40mM NaCl as in plants grown under control conditions, indicating that survival is from ionic exclusion, rather than tissue tolerance. This ability to exclude sodium breaks down at the higher concentrations, with a 100-fold increase in foliar sodium in plants grown at 80mM. This type of response is typical of plants that are sensitive to salt. Salt-tolerant species, in contrast, are able to tolerate quite high concentrations of tissue salt (Munns and Gillman, 2015). Some plants cope with excess available Na by accumulating K as a balancing cation and this may influence K^+^ uptake (Mattius, 2014; Munns and Gillman, 2015). We found no evidence for a change in ionic balance in salt-stressed cassava in the leaves. However, in the tubers, there was a significant increase in both Na and K with salt stress, though the relative increase was much greater for Na. This agrees with the results of Carretero *et al.* (2008) for roots/tubers but not leaves where it was unchanged (Carretero *et al.*, 2008), and there are also reports of potassium being lower (Hawker and Smith, 1982) than controls due to salt stress. It is not clear why there is such a difference in the effect of salinity on potassium concentration reported in these experiments, but it may be the result of plant age or that particular leaves were tested, rather than pooling material, as we did here.

While tubers are an excellent source of carbohydrates, they are very poor in nutrients (Burns *et al.*, 2010). On the other hand, leaves are an important source of protein and micronutrients for many subsistence farmers in Africa (Latif and Müller, 2015). The concentration of foliar and tuber micro- and macronutrients measured here were in the same range of concentrations as plants grown in the field in Africa (Burns *et al.*, 2012). Any change in foliar nutrients, therefore, could have a major impact on human health. In this study, there was a significant reduction in above-ground biomass in both young and old plants, and nutrient analysis of the young plants shows a significant change in important nutrients, such as iron and zinc. The concentration of these increase in leaf tissue but in terms of total nutrients available for food there is a major reduction due to salt stress. In tubers grown under moderate salt stress, there is a reduction in the concentration of macro- and micronutrients, indicating that cassava under even moderate salt stress is not a nutritious food source. There was also a small but significant decrease in foliar nitrogen concentration.

### Impact of salinity on cyanogenic glucosides and nutritional status

**Fig. 5.**
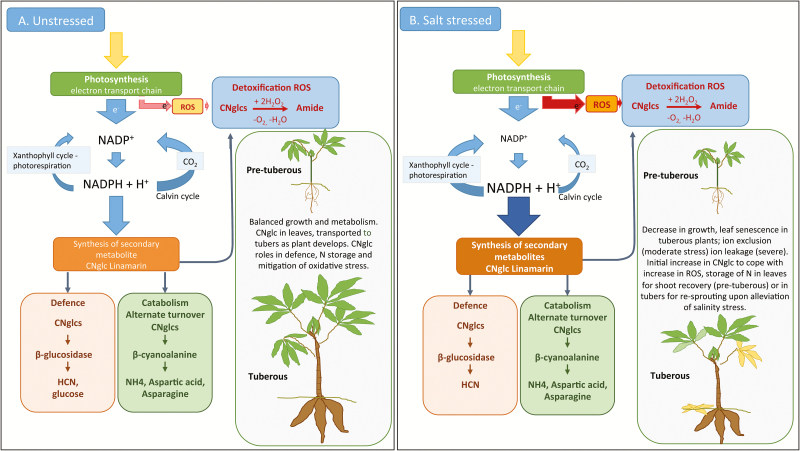
Proposed schema detailing the responses of cassava to salinity stress. During growth and development, cyanogenic glucosides (CNglcs) are synthesized in the leaves and transported to the roots and appear to play crucial roles in defence, nitrogen storage, and turnover, particularly during early plant establishment, and in mitigating oxidative stress (Jørgensen *et al.*, 2005; Selmar and Kleinwächter, 2013; Gleadow and Møller, 2014; Sendker *et al.*, 2016). (A) Under optimal growing conditions, light energy is converted to ATP and NAPDH+ and used to drive carbon fixation and other reducing reactions, such as nitrate reductase. (B) Under stress, plants shift the balance; the Calvin cycle is reduced, the xanthophyll cycle and photorespiration increase, and there is an increase in the production of secondary metabolites, such as CNglcs (Selmar and Kleinwächter, 2013. Stress enhances the synthesis of secondary plant products: the impact of stress-related over-reduction on the accumulation of natural products. Plant and Cell Physiology 54, 817–826, by permission of the Japanese Society of Plant Physiologist). The CNglcs can be turned over to release the N as ammonia or amino acids and also has been shown to be involved in the detoxification of reactive oxygen species (ROS), which also increase during stress.

Changes in the concentration of cyanogenic glucosides in our study were very different in both magnitude and direction in old and young plants. In established plants, foliar cyanogenic glucoside concentration decreased with each increment of salt from 0 to 150mM. In contrast, in pre-tuberous plants, the cyanogenic glucoside concentration was somewhat higher in the leaves of plants grown at 40mM than in those of the control plants, but much lower in plants grown at 80mM NaCl (Fig. 4A). One explanation for this difference is to consider the relative cost of the investment in defence and leaf infrastructure in the two age groups. The primary mechanism for coping with salinity stress in older plants is to discard leaves and rely on the tubers for storage, thus any investment in foliar defence is unnecessary. The significant increase in cyanogenic glucosides in the tubers of these plants is consistent with this hypothesis and also the fact that in cassava cyanogenic glucosides are synthesized in the leaves and transported to the tubers (Jorgensen *et al.*, 2005). On the other hand, in younger plants, the leaves represent a relatively large proportion of total plant biomass. Moreover, as there are no tubers, there is no sink for the storage of cyanogenic glucosides and little prospect of recovery should the shoots die. In younger plants, therefore, it is to the plant’s advantage to protect and maintain the leaves that it has already produced. Further detailed analysis of plants over time based on the hypotheses illustrated in the schematic in Fig. 5 are required.

The higher concentration of cyanogenic glucosides in the young plants from the 40mM treatment and the significant decrease in HCNp in severely salt stressed (80mM NaCl) plants may also be directly linked to the plant’s ability to tolerate stress. Salt stress promotes the production of reactive oxygen species (ROS), such as H_2_O_2_ and superoxide anions, which are toxic (Mattius, 2014; Roy *et al.*, 2014). One way to deal with stress is to have a ROS-scavenging system, for example via the up-regulation of antioxidant enzymes such as superoxide dismutase. Such enzymes are induced within minutes of salt being applied—mainly in the form of H_2_O_2_ (Cabello *et al.*, 2012; Mattius, 2014). It has been proposed that the synthesis of cyanogenic glucosides may also act to mitigate oxidative stress: NADPH^+^ is essential for the conversion of precursor amino acids to the intermediate oxime, plus the putative *in vivo* cyanogenic glucoside turnover pathway consumes H_2_O_2_ (Møller, 2010; Gleadow and Møller, 2014; Pičmanová *et al.*, 2015). The nitrogen levels in the leaves of plants at both moderate and severe salt stress do not differ significantly but the levels of HCNp do. We hypothesize that at moderate stress cyanogenic glucosides are acting in defence whilst at the higher salinity levels the cyanogenic glucosides are turned over and protect the plant from oxidative stress and ROS scavenging (Fig. 5B). However, details of the putative pathway need to be elucidated before this hypothesis can be confirmed.

### Conclusions and implications for food security

The study of climatic variables and the consequences for cassava growth is key to evaluating the effect of global change on food security. Cassava is able to tolerate a wide range of conditions and so it is often assumed that the impact of climate change on cassava will be minimal (e.g. Lobell *et al.*, 2008; Burns *et al.*, 2010). Rising sea levels and reliance on saline irrigation water in increasingly hot, dry climates poses risks that have not been previously explored for many crops.

Changes in cassava tissue chemistry in response to salinity reflect a combination of factors, including differences in the balance in trade-offs in resource allocation between growth and defence in plants at different stages of development and the possible reduction in oxygen radicals. This is summarized in the model presented in Fig. 5. This model presents a number of testable hypotheses. It is noteworthy that all the measurements of cyanogenic glucosides are above the FAO/WHO Codex (1989) standard for edible cassava flour of 10ppm cyanide, underscoring again the importance of processing before consumption

We conclude cassava to be sensitive to low to moderate concentrations of salt, particularly at early stages of development and, therefore, that cassava is not suitable for planting in regions contaminated with even relatively low levels of salt. In coastal areas, impacts may be minimized by irrigating with less saline water, or during periods of high rainfall to allow time for plants to become established before they are exposed to higher concentrations of salt. Given that alternative tuberous crops such as sweet potatoes are even more salt sensitive than cassava (Shannon and Grieve, 1999), breeding for more salt-tolerant varieties is necessary if cassava is to continue to expand its role as a staple in a future, more saline world.

## Supplementary data

Supplementary data are available at *JXB* online.

**Table S1.** Height, leaf area, biomass, growth indices, and chlorophyll measurements of cassava plants grown at three levels of salinity (0, 40, and 80mM NaCl) for 70 d.

**Figure S1.** Total biomass of 6-month-old, tuberous cassava plants after 4 weeks of treatment with different concentrations of salt (Experiment 1).

**Figure S2.** Total biomass of young, pre-tuberous cassava plants plotted versus salinity (Experiment 2).

**Figure S3.** Correlation between total chlorophyll and greenness measured using the GreenIndex Ap.

Supplementary Data

## Abbreviations:

HCNp: hydrogen cyanide potential
NaCl: sodium chloride
RGR: relative growth rate
SLA: specific leaf area.
