# Supplementary material for: Resilience of cassava (Manihot esculenta Crantz) to salinity: implications for food security in low-lying regions
Source: J Exp Bot. 2016 Aug 9;67(18):5403–13. doi: 10.1093/jxb/erw302 (PMC5049390; doi:10.1093/jxb/erw302)
Supplement: Supplementary Data [file supp_67_18_5403__index.html]

Resilience of cassava (Manihot esculenta Crantz) to salinity: implications for food security in low-lying regions — Resilience of cassava (Manihot esculenta Crantz) to salinity: implications for food security in low-lying regions — Supplementary Data 

# Resilience of cassava (*Manihot esculenta* Crantz) to salinity: implications for food security in low-lying regions

## Supplementary Data

Data files

- supplementary\_table\_S1\_figures\_S1\_S3.pdf - Supplementary Data
