# Supplementary material for: Resilience of cassava (Manihot esculenta Crantz) to salinity: implications for food security in low-lying regions
Source: J Exp Bot. 2016 Aug 9;67(18):5403–13. doi: 10.1093/jxb/erw302 (PMC5049390; doi:10.1093/jxb/erw302)

Supplementary Data

Table S1. Height, leaf area, biomass, growth indices and chlorophyll measurements of cassava plants grown at three levels of salinity (0, 40 and 80mM NaCl) for 70 days. Plants were approx. 2 months old at the start of the experiment. Values are means (±1SE) of 10 replicates.

	**0 mM NaCl**	**40 mM NaCl**	**80 mM NaCl**	**Significance**
**Stem height (cm)**	22.31±0.49	20.82±0.97	11.29±0.89	P < 0.0001
**Leaf number**				
Unexpanded leaves	3.1±0.2	2.9±0.4	0.5±0.3	P < 0.0001
Expanded leaves	8.6±0.4	6.8±0.6	0.7±0.4	P < 0.0001
Senescent leaves	2.1±0.4	3.2±0.5	4.7±0.6	P = 0.006
**Area (cm^2)**				
Unexpanded leaves	144.4±19.3	115.1±11.4	10.0±6.1	P < 0.0001
Expanded leaves	689.1±33.64	354.2±31.2	22.8±13.9	P < 0.0001
Senescent leaves	144.5±5.0	115.1±1.4	43.3±9.8	P < 0.0001
**Biomass (g dry wt)**				
Cutting stock*	1.197±0.118	1.119±0.147	1.047±0.193	P = 0.795
Above ground mass	3.653±0.191	2.475±0.200	0.628±0.140	P < 0.0001
Stem plus petiole	1.491±0.097	1.073±0.095	0.216±0.032	P < 0.0001
Total leaf mass	2.162±0.096	1.402±0.109	0.412±0.125	P < 0.0001
Unexpanded leaves	0.382±0.046	0.334±0.031	0.027±0.016	P < 0.0001
Expanded leaves	1.611±0.087	0.863±0.085	0.172±0.138	P < 0.0001
Senescent leaves	0.169±0.040	0.205±0.034	0.213±0.040	P=0.680
Below ground mass	4.032±0.284	3.258±0.254	1.462±0.266	P < 0.0001
Tuberous roots	7.685±0.432	5.733±0.416	2.090±0.322	P < 0.0001
Fine roots	2.488±0.231	1.962±0.218	0.410±0.113	P < 0.0001
**Growth indices**				
Root:Shoot	1.11±0.06	1.33±0.08	2.98±0.64	P=0.0024
SLA cm^2/g	430.0±7.9	413.9±7.2	n/a	P=0.77
RGR g/g/day	0.88±0.024	0.75±0.035	0.27±0.06	P < 0.0001
**Chlorophyll, Greenness**				
Greenness#	0.80±0.03	0.75±0.03	0.45±0.10	P < 0.0001
Total chlorophyll	66.12±1.13	59.44±1.37	8.68±1.84	P < 0.0001
Chlorophyll a:b	0.83±0.03	1.09±0.06	1.12±0.10	P=0.0037

* Mass of the cutting taken from the parent plant. This continues to grow after the plants develop.
# Greenness was measured visually using the GreenIndex+ Ap

Supplementary Figures

There are three supplementary figures.

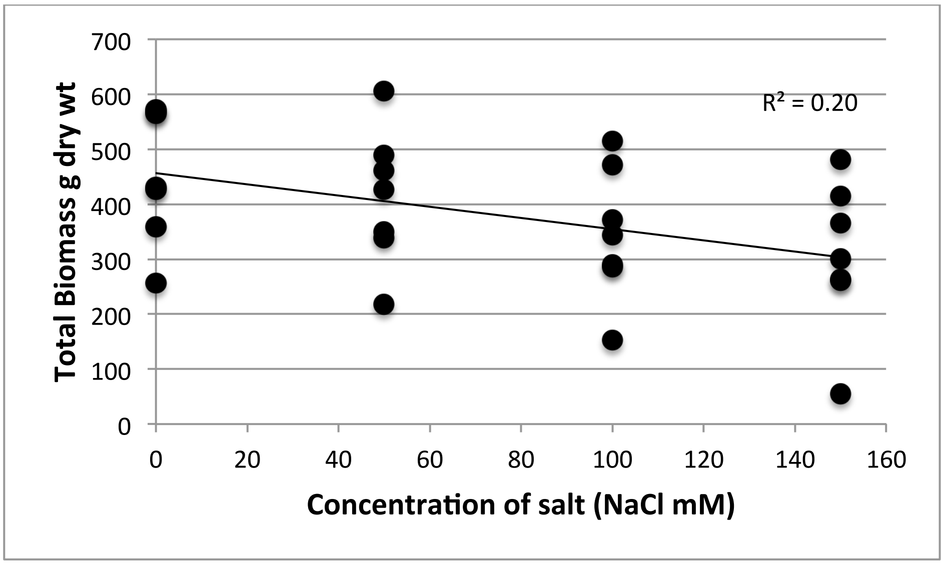

Fig. S1. Total biomass of 6 month old, tuberous cassava plants after 4 weeks of treatment of with different concentrations of salt (Experiment 1). The regression equation (y = -1.0197x + 456.6) is significant with a correlation coefficient of R^2=0.20 (P=0.02)

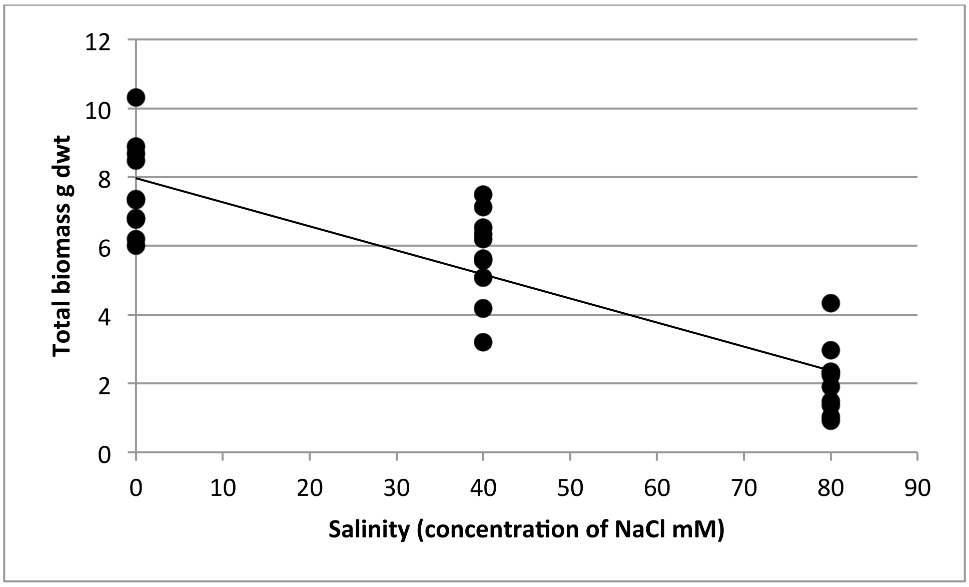

Fig. S2. Total biomass of young, pre-tuberous cassava plants plotted versus salinity (Experiment 2). The regression equation (y = -0.069x + 7.967, R^2 = 0.77) is significant (P<0.0001).

Fig. S3. Correlation between Total Chlorophyll and Greenness measured using the GreenIndex Ap. Measurements were made on the third fully expanded leaf of cassava grown at three concentrations of salt. The correlation is significant ($P<0.001$) with an R2 of 0.45 and a Pearson's correlation coefficient of 0.67. The regression equation is *y = 81.99x - 7.052.*

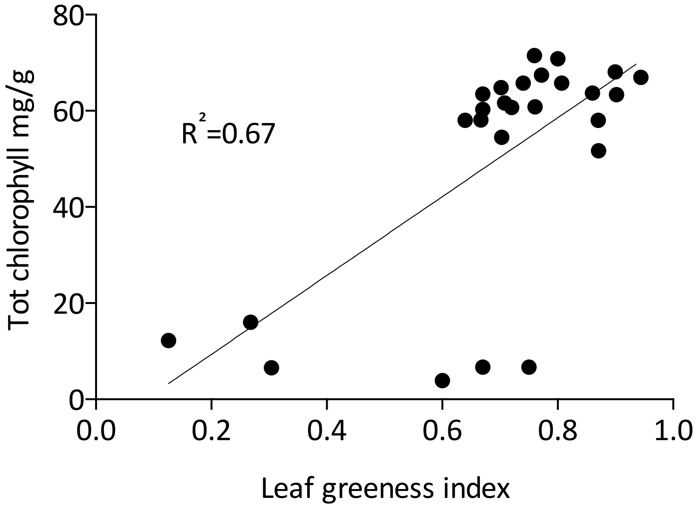

Supplement: Supplementary Data [file supp_erw302_supplementary_table_S1_figures_S1_S3.pdf]
